# Human umbilical cord mesenchymal stromal cell-derived exosomes protect against MCD-induced NASH in a mouse model

**DOI:** 10.1186/s13287-022-03201-7

**Published:** 2022-11-12

**Authors:** Ying Shi, Xiaoguang Yang, Shuyue Wang, Yulun Wu, Lihua Zheng, Yufang Tang, Yanhang Gao, Junqi Niu

**Affiliations:** 1 Hepatology Department, First Hospital of Jilin University, No. 1, Xinmin Street, Changchun, 130000 Jilin People’s Republic of China; 2 National Engineering Laboratory for Druggable Gene and Protein Screening, Northeast Normal University, 2555 JingYue Street, Changchun, 130000 Jilin People’s Republic of China; 3 Biomedical Laboratory Center of Wish Vocational Training School, Beihu Science and Technology Zone, Changchun, 130012 Jilin People’s Republic of China

**Keywords:** Nonalcoholic steatohepatitis, hUC-MSC exosomes, PPARα, MCD mouse model

## Abstract

**Background and aims:**

Human umbilical cord mesenchymal stem cells (hUC-MSCs) are increasingly being studied in clinical trials of end-stage liver disease because of their good tissue repair and anti-inflammatory effects. hUC-MSC exosomes are vesicles with spherical structures secreted by cells that produce them. The diameter of exosomes is much smaller than that of hUC-MSCs, suggesting that exosomes might be a novel and safer therapeutic product of mesenchymal stem cells. As exosomes have been suggested to have biochemical functions similar to those of hUC-MSCs, this study investigated the efficiency of hUC-MSC-derived exosomes in protecting against nonalcoholic steatohepatitis using an MCD-induced mouse model.

**Methods:**

Human umbilical cord mesenchymal stem cell-derived exosomes were extracted and purified. The effect of these exosomes on disease progression in an MCD-induced nonalcoholic steatohepatitis mouse model was investigated.

**Results:**

The results showed that UC-MSC exosomes intravenously transplanted into mice with MCD-induced NASH improved MCD-induced body weight loss and liver damage in a mouse model. Additionally, the inflammatory cytokines in liver tissue were reduced, which may be caused by exosome-induced macrophage anti-inflammatory phenotypes both in vitro and in vivo. In addition, UC-MSC exosomes reversed PPARα level in ox-LDL-treated hepatocytes in vitro and in NASH mouse liver, which had been downregulated.

**Conclusions:**

UC-MSC exosomes alleviate MCD-induced NASH in mice by regulating the anti-inflammatory phenotype of macrophages and by reversing PPARα protein expression in liver cells, which holds great potential in NASH therapy.

**Supplementary Information:**

The online version contains supplementary material available at 10.1186/s13287-022-03201-7.

## Introduction

Human umbilical cord mesenchymal stem cells (hUC-MSCs) can be cultured not only in vitro but also in abundant amounts. hUC-MSCs have stable characteristics and low immunogenicity and have been clinically used in the treatment of cardiovascular, Parkinson’s and cirrhosis diseases. The paracrine effect of MSCs has been indicated in recent research, both in preclinical and clinical studies [1–4]. MSCs can synthesize and secrete a broad spectrum of chemokines and growth factors, which exert significant effects on repairing injured tissues, enhancing angiogenesis and protecting against ischemic renal tissue [4–6]. Exosomes derived from hUC-MSCs are vesicles 40–150 nm in diameter that contain contents similar to UC-MSCs, including mRNAs, microRNAs, DNAs and proteins. It has been suggested that exosomes have biochemical functions similar to those of MSCs in terms of wound repair, tissue regeneration, anti-apoptosis effects and inflammation factor inhibition in vitro [7, 8]. Recently, with skilled vesicle preparations, in addition to their small size, low immunogenicity, and convenient transport and storage, exosomes have been tested in many animal models of disease in preclinical efficacy studies [9–12]. Notably, exosome treatments have shown results very similar to MSC transplantation while avoiding many risks. However, little is known about the efficiency of umbilical cord MSC exosomes in improving nonalcoholic steatohepatitis (NASH).

Nonalcoholic fatty liver disease (NAFLD) is a metabolic disease caused by multiple factors. Progressive NAFLD may result in nonalcoholic steatohepatitis. Hepatocytes begin with steatosis and inflammation, leading to liver fibrosis and end-stage liver disease [13–15]. Currently, NASH therapy mainly involves lifestyle intervention with drugs that improve lipid metabolism, insulin sensitivity, oxidation, and fibrosis. To date, no pharmacotherapy other than aubercholic acid has been approved for NASH as a breakthrough use for NASH treatment [16–18]. Therefore, new therapeutic options for NASH are still awaiting development. Recently, MSCs have been reported to effectively inhibit T lymphocyte differentiation in a NAFLD mouse model by decreasing high-fat diet-induced weight gain, steatosis and lobular inflammation [19]. Additionally, Li et al. [20] found that UC-MSC infusions could ameliorate hyperglycemia and liver injury in a mouse model of nonalcoholic fatty liver disease accompanied by type 2 diabetes, indicating that MSCs may be considered a new strategy for NAFLD. However, there are still some unsolved problems reported in MSC preclinical efficiency experiments; for example, only < 1% of transplanted MSCs could localize in the target tissue, while most cells were trapped in the lungs [21], leading to a 25–40% higher incidence of pulmonary embolism and death when MSCs were infused by intra-arterial administration [22]. A new study elucidated that adipose tissue-derived MSCs and their small extracellular vesicles attenuated inflammation and reduced fibrosis in a rapid NASH fibrosis model, indicating that mesenchymal stem cell exosomes and MSCs may have similar functions [23]. However, compared with MSCs, MSC exosomes may have fewer side effects and safer infusion methods.

In this study, we purified umbilical cord mesenchymal stem cell exosomes, investigated the effects of these exosomes on liver biochemical indices, pathology, and PPARα in mice with MCD-induced nonalcoholic steatosis and discovered the potential role of umbilical cord mesenchymal stem cell exosomes in improving the treatment of nonalcoholic steatosis.

## Materials and methods

### Reagents

Serum-free additive was purchased from Elitecell Biomedical Corp. (EPA-050, Texas, USA), complete medium was purchased from Darkway Biotechnology Co., Ltd. (6,114,021, 6,114,541, 6,114,551, 6,114,531, Beijing, China), PBS was purchased from HyClone Co. Ltd. (SH30406.05IR30-40, South Logan, UT, USA), and trypsin was purchased from Gibco Invitrogen Co. (New York, NY, USA). PKH26 was purchased from Sigma–Aldrich (PKH26PCL, San Francisco, CA, USA). Oxidized low-density lipoprotein (Ox-LDL) was purchased from Yiyuan Biotechnology (YB-001, Guangzhou, China). The primary antibody targeting F4/80 was purchased from Abcam (ab100790, Cambridge, UK). Antibody for FACS includes PE anti-human CD73 (clone AD2, Biolegend), FITC anti-human CD90 (clone 5E10, Biolegend), PE anti-human CD105 (clone 266, BD Pharmingen), FITC anti-human CD19 (clone HIB19, Biolegend), FITC anti-human CD34 (clone 581, Biolegend), FITC anti-human CD14 (clone M5E2, Biolegend) and FITC Mouse IgG1, κ (clone MOPC-21, Biolegend) PE Mouse IgG1, κ (clone MOPC-21, Biolegend) as isotype control. For Western blot, the primary antibody targeting both human and mouse PPARα was purchased from Boster Biological Technology Co. Ltd. (BA1691, Wuhan, China). The primary antibodies targeting histone H3, CD63, TSG101, CD9, GAPDH and alkaline phosphatase-conjugated secondary antibodies (SA00002-1 and SA00002-2) were purchased from Proteintech (Chicago, IL, USA). Cell lysis buffer and DAPI were purchased from Beyotime (Beijing, China). All enzyme-linked immunosorbent assay (ELISA) kits were purchased from R&D Systems (Minneapolis, MN, USA). The methionine–choline-deficient (MCD) diet (MD12052) and control diet were purchased from Trophy Feed Technology Co., Ltd. (Jiangsu, China). The methionine and choline contents in the MCD feed are close to 0, and the fat content is 7%. The control feed had methionine and choline contents within the reference standard level and was otherwise matched with the model feed.

### Human umbilical cord mesenchymal stromal cell (hUC-MSC) isolation and culture

The experimental hUC-MSCs were preserved in the International Joint Research Center of the National Human Stem Cell Bank. The cells were cultured to passages 4–7.

### Identification of MSC adipogenic and osteogenic differentiation

Cells were seeded into 24-well culture plates at a density of 4 × 10^4^ cells/well. After 2–3 days of culture, the growth of cells was observed. The lipid induction medium or osteogenic induction culture medium was replaced in the experimental cells, and the complete medium was replaced in the control cells. The 24-well plate was incubated in a CO_2_ incubator at a stable temperature, and the liquid medium was changed every 2–3 days. The growth, differentiation and morphological characteristics of the cells were observed. On the 16th day of induction culture, the cells were stained with either oil red O or Alizarin red, observed under an inverted microscope and photographed.

### Identification of MSC differentiation into cartilage

The digested and collected cells were inoculated into a 15-mL centrifuge tube at a density of 2.5 × 10^5^ cells/tube and centrifuged at 300 g at room temperature for 5 min. After forming microspheres, they were transferred to a 24-well cell culture plate. The chondrocyte induction medium was changed in the experimental wells. The liquid was changed every 2–3 days, and the growth, differentiation and morphological characteristics of the cells were observed. On the 21st day of induction culture, the tissue was stained with toluidine blue and washed once with PBS. Frozen sections of the tissue spheres were stained with toluidine blue for 5 min, washed with pure water twice, observed under an inverted microscope and photographed.

### Preparation of exosomes

hUC-MSCs at passages 4–7 were cultured to 90% confluence in 10-cm-diameter dishes. The adherent cells were incubated in 5% vesicle-depleted fetal bovine serum (FBS) for at least 24 h. Then, the conditioned medium was collected and centrifuged at 4 °C at 2000 g for 10 min to remove the cells and at 10,000 g for 5 min to remove debris. hUC-MSC-derived exosomes were extracted using an exoEasy Maxi Kit (Qiagen, Hilden, Germany) following the manufacturer’s instructions. The morphology of MSC exosomes was examined using transmission electron microscopy (TEM). The expression of CD9, Tsg101 and CD63 was confirmed by western blot.

### Cell culture

HepRG cells were obtained from the Shanghai Institute of Biochemistry and Cell Biology (Shanghai, China). HepRG cells were cultured in RPMI 1640 (72,400,120, Gibco, USA) supplemented with 10% FBS (04-001-1A, BI, Israel). The mouse liver cell line Huh1-6 was preserved in our laboratory, and the Raw264.7 mouse macrophage line was a gift from the Institute of Immunization, Jilin University. Huh1-6 and RAW264.7 cells were cultured in DMEM (01-052-1ACS, BI, Israel) supplemented with 10% FBS (04-001-1A, BI, Israel). All cells were incubated at 37 °C with 5% CO_2_.

### Animal treatment

C57BL/6 J mice (male) with body weights of 22–25 g were purchased from Vital River Laboratories (Beijing, China). All mice were maintained under standard light conditions (12-h light/dark cycle) at room temperature. After 7 days of adaptive feeding, the mice were randomly divided into four groups. Group 1 was fed regular chow (wild-type). Group 2 was the disease model group which were fed a methionine–choline-deficient (MCD) diet without any intervention. In the other two groups, the mice were fed an MCD diet and received tail vein injections once a week of either conditioned medium extract (using an exosome preparation protocol) or exosomes. Each mouse was injected with 20 mg/kg body weight hUC-MSC-derived exosomes twice a week. MCD feeding lasted for 6 weeks. At the end of the experiment, animals were injected intraperitoneally with pentobarbital sodium (1% v/v) for anesthesia. Peripheral blood was collected and centrifuged to obtain serum for biochemical analyses and cytokine measurements. The fresh liver was excised and stored at − 80 °C for further use. Fixed tissues in each group were used to prepare pathological sections. The protocol was performed as indicated. All procedures were in accordance with the Institutional Guidelines for Animal Experiments.

### Histological analysis

Liver samples from each mouse were fixed in 10% (v/v) formalin and then dehydrated in a series of gradient alcohol washes. The tissues were cleared in xylene and embedded in paraffin. Sections were approximately 3 μm and stained with hematoxylin and eosin (H&E). The levels of F4/80 and NF-κB(p65) in the tissues were analyzed by immunohistochemistry as previously described [24]. For the histological diagnosis of NASH, the NAFLD activity score was evaluated by a pathologist using standards as previously described [25].

### Biochemical analyses

The levels of alanine transaminase (ALT) and aspartate aminotransferase (AST) in the serum were measured by a Roche Cobas testing assay.

### Western blot analysis

Both tissue and cell protein concentrations and extracted exosomes were detected using a BCA assay kit. The lysates were separated by 12% (w/v) SDS–PAGE. After electrophoresis, the proteins were transferred to nitrocellulose (NC) membranes and blocked with 5% (w/v) bovine serum albumin (BSA) in phosphate-buffered solution for 1 h at room temperature. The membranes were probed with the indicated primary antibodies for 4 h at room temperature, washed three times and incubated with AP-conjugated secondary antibodies for 1 h at room temperature. The NC membranes were washed with PBST (PBS with 0.5% Tween-20) three times after each hybridization step. Protein levels were detected by a BCIP/NBT alkaline phosphatase color development kit.

### Enzyme-linked immunosorbent assay (ELISA)

Inflammatory cytokine levels in plasma were measured using ELISA kits (R&D) specific for mouse TNF-α, IL-1 and IL-6, according to the manufacturer’s instructions. The absorbance of the plates was read at 450 nm. The cytokine levels were measured and calculated by standard curves prepared with the respective recombinant cytokines.

### Quantitative real-time polymerase chain reaction

Total RNA was extracted from mouse liver tissue, and single-stranded cDNA was synthesized with a reverse transcription kit (Takara Bio, Kusatsu, Japan). Real-time quantitative polymerase chain reaction (PCR) analysis was conducted with SYBR Green PCR master mix (Takara Bio, Kusatsu, Japan). GAPDH was used as a reference gene in each sample. All primers were purchased from Sangon Biotech Co., Ltd. (Shanghai, China). The primers are presented in Table 1.

**Table 1:** The primer name and the sequences

	Forward	Reverse
*IL-1β*	TGGACCTTCCAGGATGAGGACA	GTTCATCTCGGAGCCTGTAGTG
*TNF-a*	GGTGCCTATGTCTCAGCCTCTT	GCCATAGAACTGATGAGAGGGAG
*IL-6*	TACCACTTCACAAGTCGGAGGC	CTGCAAGTGCATCATCGTTGTTC
*CD206*	GTTCACCTGGAGTGATGGTTCTC	AGGACATGCCAGGGTCACCTTT
*Arg-1*	CATTGGCTTGCGAGACGTAGAC	GCTGAAGGTCTCTTCCATCACC
*IL-10*	CGGGAAGACAATAACTGCACCC	CGGTTAGCAGTATGTTGTCCAGC
*GAPDH*	CATCACTGCCACCCAGAAGACTG	ATGCCAGTGAGCTTCCCGTTCAG

### Statistical analysis

Statistical analyses were performed by Prism software (GraphPad 5.0). Student’s t test was performed to compare experimental and relative control groups. Statistical p values < 0.05 were considered significant, and *p* values < 0.01 were considered extremely distinct. Statistical p values > 0.05 were considered nonsignificant (NS).

## Results

### Characterization of hUC-MSCs and MSC exosomes

**Fig. 1:**
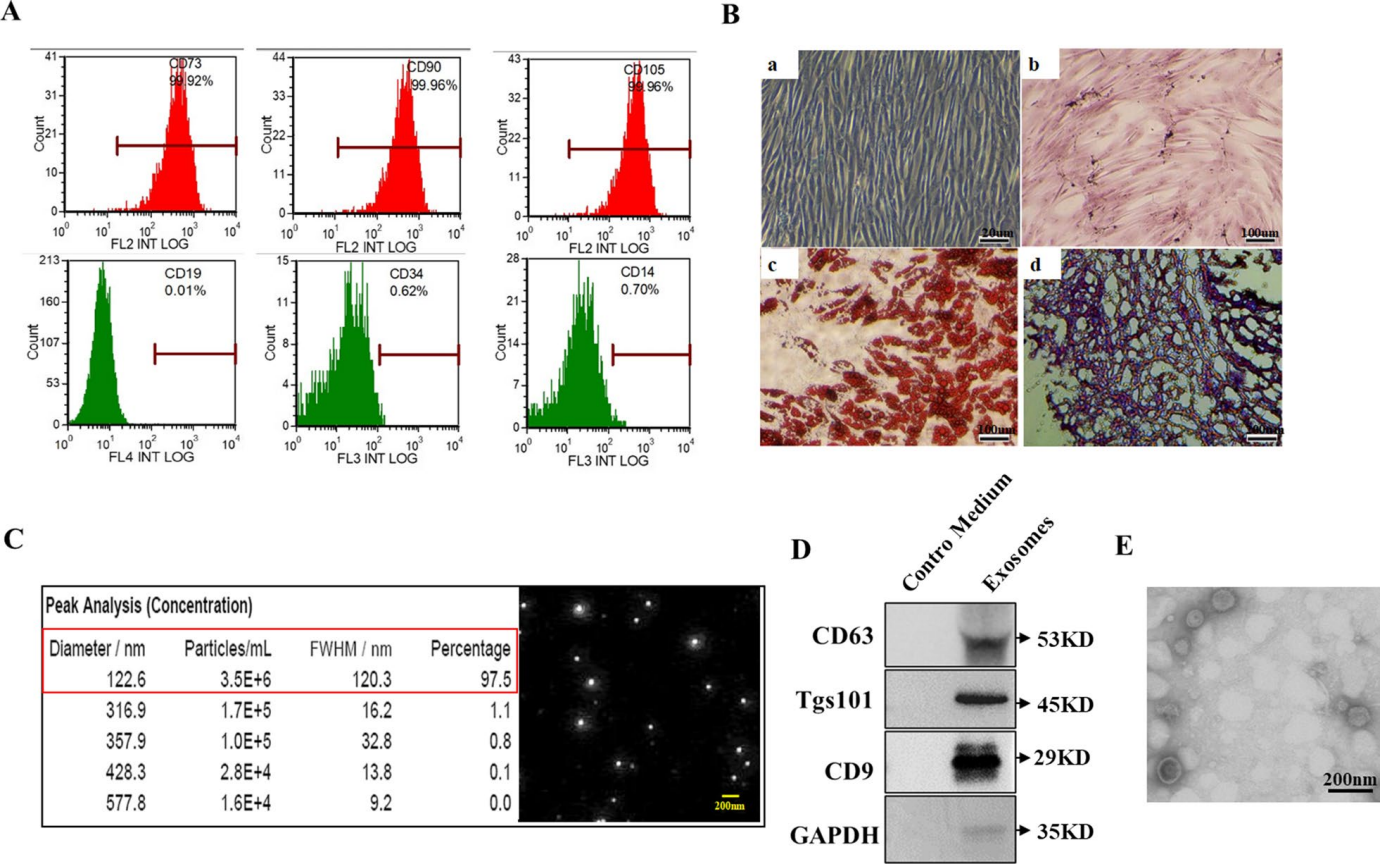
**A**. MSC surface marker expression analysis by flow cytometry. **B**. (a) Fifth-generation hUC-MSCs under a light microscope; (b and c) osteogenic and adipogenic differentiation of hUC-MSCs; (d) cartilage formation differentiation of hUC-MSCs. **C**. The vesicle diameter, number, purity, and morphology were tested by electrophoresis (red box, left panel) and Brownian motion video analysis laser scattering microscopy (right panel). **D**. Western blot detection of the expression of TS101, CD63, CD9, and GAPDH in hUC-MSC-derived exosomes. The negative control was the product extracted from the control medium (CM) according to exosome extraction steps. Full-length blots are presented in Additional file 1: Fig. S1. **E**. Transmission electron micrographs of hUC-MSC exosomes. Scale bar = 200 nm

To obtain hUC-MSC exosomes, hUC-MSCs were identified by flow cytometric analysis. As shown in Fig. 1A, the majority of hUC-MSCs expressed high levels of CD90, CD105 and CD73, whereas CD34, CD45 and CD19 positive cells were present in very low proportions. Figure 1B shows the morphology of fifth-generation hUC-MSCs under a light microscope (a) and the ability of these hUC-MSCs to differentiate into osteocytes, adipocytes and cartilage (b, c and d). After collection and isolation, the vesicle diameter, number, purity, and morphology of hUC-MSC exosomes were evaluated by nanoparticle tracking analysis (NTA) and western blotting, as shown in Fig. 1C and D. The NTA data showed that more than 95% of the vesicles were 120 nm in diameter. The concentration of hUC-MSC exosomes was 4.4 × 10^10^ particles/ml. Western blot detection of the specific marker TS101, CD63, CD9, and GAPDH expression in hUC-MSC-derived exosomes is shown in Fig. 1D. The transmission electron micrographs of hUC-MSC exosomes are shown in Fig. 1E.

### UC-MSC exosomes protect against MCD-induced body weight loss and liver damage in a mouse model

**Fig. 2:**
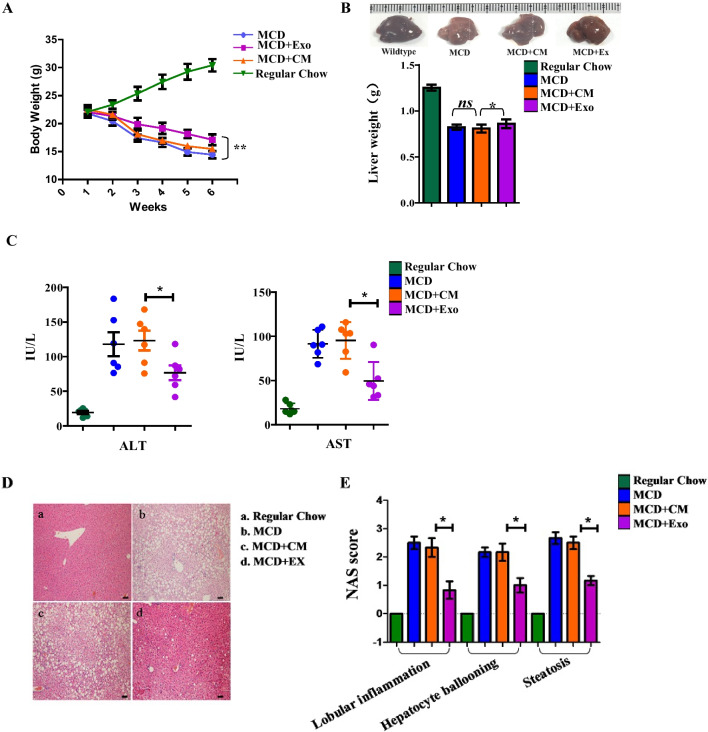
**A**. Weight changes of mice fed regular chow or MCD during 6 weeks period. In each group, the number of mice was n = 6. **p < 0.01. **B**. Photographs of liver sizes of mice from different experimental groups (upper panel). The weights of each group of mice were recorded and counted (n = 6, below panel). **p < 0.01. *ns*: not significant. **C**. Plasma levels of alanine aminotransferase (ALT) and aspartate aminotransferase (AST) of each mouse were recorded and counted (n = 6 in each group); *p < 0.05; **D**. Representative images of HE-stained liver sections of the indicated groups (microscope 40×). a mice with regular chow; b mice with MCD; c mice with MCD and intervention with control medium (CM) extract twice a week; d mice with MCD and intervention with hUC-MSC exosomes(Ex) twice a week. Scale bar = 50 μm. **E**. Pathology scores for balloon-like degeneration, steatosis, and lobular inflammation of groups of liver sections (n = 6). *p < 0.05, **p < 0.01

After 6 weeks of disease model induction, no death occurred in the experimental mice. The body weight of mice in each group during MCD induction was recorded. The baseline mouse body weight in each group was almost even, as shown in Fig. 2A. The body weight of mice fed regular chow increased nearly 40% over 6 weeks, while in the MCD groups, the body weights decreased 25% over 6 weeks. The mice in the UC-MSC exosome intervention group also continually lost weight, which was moderate compared to the weight loss of the MCD group or those administered vein injection with control medium extract (MCD + CM). At week 6, mice treated with exosomes showed significant differences in body weight compared with those in the MCD + CM group (*p* < 0.01). Next, the mouse livers were separated and weighed. As shown in Fig. 2B, compared with the livers of mice on regular chow, the MCD diet caused liver atrophy and weight loss, which could be improved by exosome intervention (*p < 0.05). The ratio of liver to body weight in each group was also calculated, but no significant difference could be obtained. AST and ALT were measured to determine hepatocellular injury in these groups of mice. As shown in Fig. 2C, exosome intervention significantly reduced AST and ALT compared to the levels of the other MCD feeding groups (*p < 0.05), suggesting that UC-MSC exosomes effectively reduce liver damage in the MCD-induced NASH model. Histological staining indicated that UC-MSC exosomes alleviated steatosis compared to those of the other MCD feeding groups (Fig. 2D), and pathology scores for balloon-like degeneration and lobular inflammation were also reduced (Fig. 2E). These results suggest that UC-MSC exosomes could protect against MCD-induced body weight loss and liver damage and have a potential role in enhancing lipid metabolism in MCD-induced mouse NASH development.

### Exosomes reduced inflammatory cytokines in liver tissue and increased macrophage anti-inflammatory activity

**Fig. 3:**
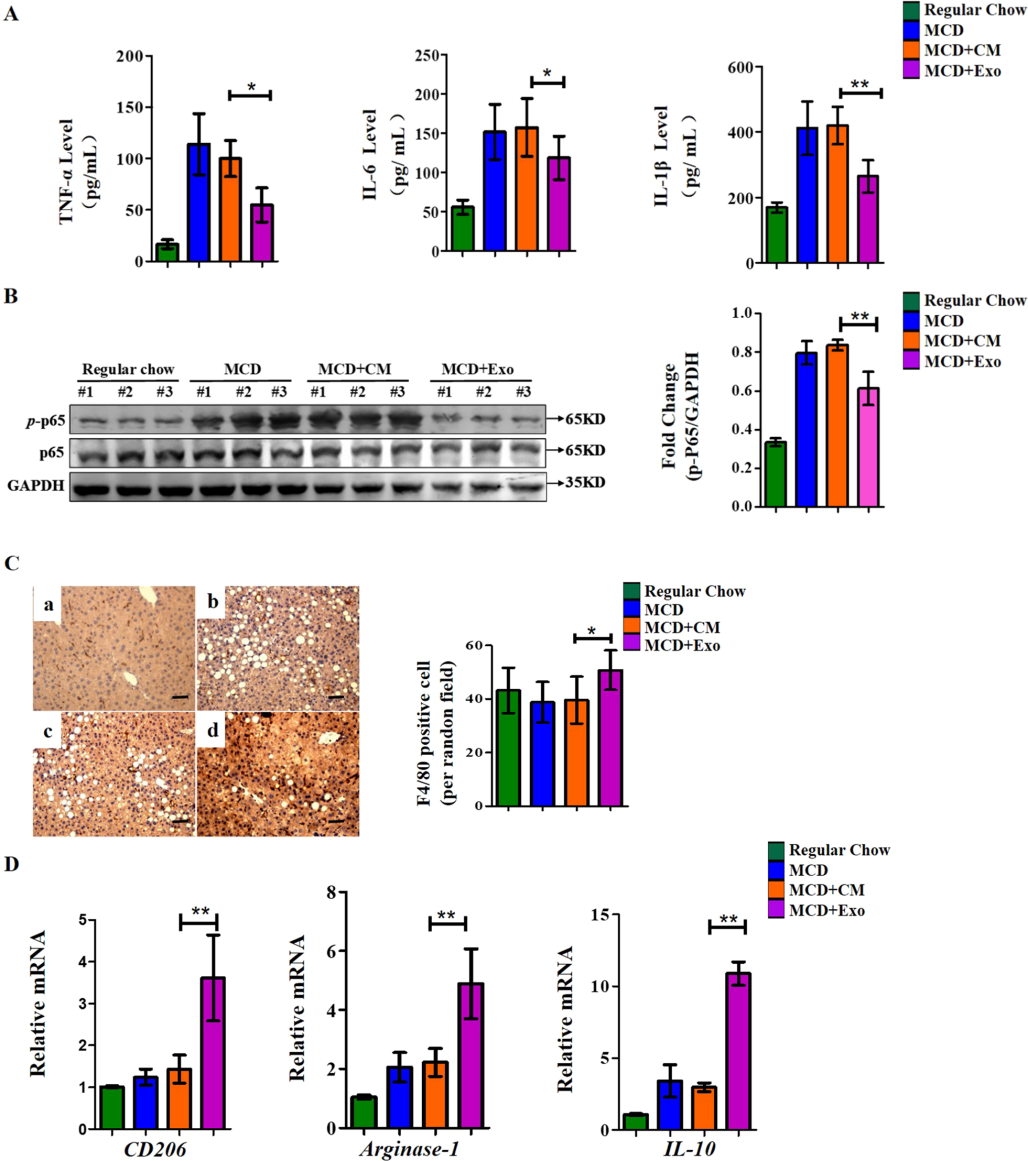
**A** Plasma levels of TNF-α, IL-6, and IL-1β in groups of mice, n = 6. Protein levels were evaluated using the sandwich ELISA method. *p < 0.05. **B**. Western blotting analysis of phosphorylated NF-κB (*p*-P65) and total NF-κB (P65) in liver tissues in groups of mice (left panel). Three liver tissues were randomly selected from each group to compare NF-κB activation levels. GAPDH protein levels were used as a reference standard. The phosphorylated NF-κB protein was been quantified using ImageJ software and represented by histogram (fold change of phosphorylated NF-κB /GAPDH, right panel). Full-length blots are presented in Additional file 1: Fig. S2. **C**. Immunohistochemical assay targeting F4/80 was used to detect macrophages in groups of mouse livers. (a) Regular chow; (b) MCD group; (c) MCD with control medium (CM) extract intervention group; (d) MCD with hUC-MSC exosomes (Ex) group (left panel). Scale bar = 50 μm. Three fields were randomly selected to determine the number of macrophages in the livers of mice in each group, and the differences among groups were compared (right panel). *p < 0.05. **D**. Quantitative PCR results showing the endogenous mRNA levels of CD206, Arginase-1, and IL-10 in mouse livers in each group (n = 3). GAPDH mRNA served as reference standard. The fold change is shown by the histogram. **p < 0.01

According to the histological changes, we further examined inflammatory cytokine production levels in the plasma of each group of mice. As shown in Fig. 3A, consistent with the histology results, mice with MCD had significantly upregulated TNF-α, IL-6 and IL-1β in plasma. However, with UC-MSC exosome administration, these inflammatory cytokines were significantly reduced compared to those in the other MCD groups. It has been suggested that the transcription of inflammatory cytokines is mediated by phosphorylation of NF-κB(p65), which is shown in Fig. 3B. The phosphorylation of NF-κB(p65) protein level in the liver was significantly increased in MCD-fed mice compared to that in wild-type mice, while exosome intervention could partly reverse this increase, which was the same variation trend as the inflammatory cytokine production in the liver. However, the total NF-κB level was also changed by different treatments. Furthermore, as macrophages are the main source of inflammatory cytokines in the liver, an F4/80 antibody was used to mark the number of macrophages in the liver of mice in each group. As shown in Fig. 3C, compared with other MCD mice, more macrophages were recruited to the livers of mice in the exosome intervention group, indicating that exosomes potentially altered the distribution and chemotaxis of macrophages in the injured liver. In addition, total mRNA from three mouse liver tissues in each group was extracted, and the levels of markers of the anti-inflammatory phenotype of macrophages, namely, *CD206, Arginase-1* and *IL-10*, were detected by quantitative PCR. As shown in Fig. 3D, compared with the normal diet in mice, the transcription levels of *CD206, Arginase-1* and *IL-10* were all increased. However, these three macrophage markers were significantly enhanced in the livers of exosome-treated mice, indicating that exosomes may increase the number of anti-inflammatory macrophages or promote macrophage transformation to an anti-inflammatory phenotype in MCD-induced liver damage.

### *Exosomes reduced ox-LDL-induced macrophage inflammatory phenotypes *in vitro

**Fig. 4:**
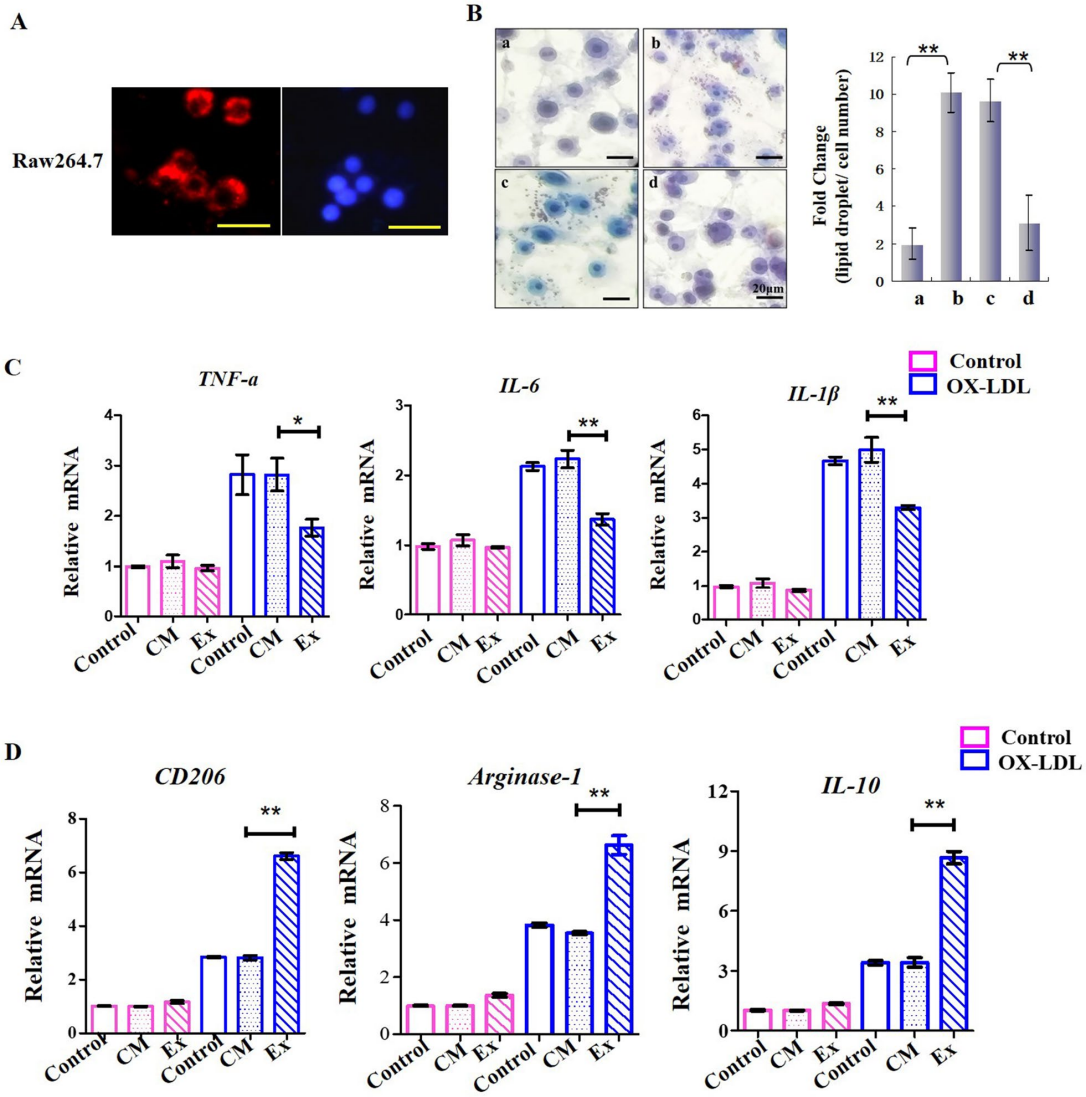
**A**. PKH-26 labeling of hUC-MSC exosomes taken up by RAW264.7 cells. Scale bar = 20 μm. **B**. Oil red O staining of foamy RAW264.7 cells (left panel). (a) Without any treatment; (b) Ox-LDL treatment for 48 h; (c) Ox-LDL treatment for 48 h after control medium (CM) extract treatment for 24 h; d. Ox-LDL treatment for 48 h after UC-MSC treatment for 24 h. Three fields were randomly selected to calculate the number of red lipid droplets and the number of cells. The fold change is shown by the histogram (right panel). **C**. Quantitative PCR results showing the endogenous mRNA levels of *TNF-α, IL-6* and *IL-1β* in RAW264.7 cells. CM represents control medium extract treatment, and Ex represents UC-MSC exosome treatment. The independent experiment has been repeated for three times. *p < 0.05; **p < 0.01. **D**. Detection of the M2 polarization markers *CD206, Arginase-1* and *IL-10* in Ox-LDL-treated RAW264.7 cells by quantitative PCR. CM represents control medium extract treatment, and Ex represents UC-MSC exosome treatment. GAPDH mRNA served as reference standard. *p < 0.05; **p < 0.01

In NAFLD, cholesterol metabolism disorder causes macrophage foaming and the release of a large amount of inflammatory factors, which are considered an important source of inflammatory cytokines for maintaining NASH. To investigate whether exosomes could reduce macrophage foaming and the release of inflammatory factors, the ability of the mouse macrophage cell line RAW264.7 to take up anthropogenic exosomes was tested. As shown in Fig. 4A, PKH26 fluorescently labeled exosomes were cocultured with RAW264.7 cells, and 1 h later, exosomes could be taken up by these cells and emitted fluorescence. To identify whether exosomes could influence macrophage foaming and the transcription of inflammatory factors in foaming macrophages, RAW264.7 cells were treated with oxidized low-density lipoprotein (ox-LDL) for 48 h, before which time two groups of cells had been treated with control medium extract or UC-MSC exosomes for 24 h. Then, foam cells were detected by oil red O staining (Fig. 4B), and the expression levels of *IL-6, TNF-α* and *IL-1β* were evaluated by real-time PCR (Fig. 4C). As shown in Fig. 4B, ox-LDL treatment significantly increased the number of lipid droplets in RAW264.7 cells, which could be reversed by exosome treatment. In the right panel of Fig. 4B, the histogram shows the ratio of the lipid droplets to the quantity of cells in each field of view. Exosomes also downregulated the ox-LDL-induced production of three inflammatory factors (*TNF-α, IL-6* and *IL-1β*) by RAW264.7 macrophages, which could be visibly reversed by UC-MSC exosome treatment (Fig. 4C). The expression levels of molecular markers of the anti-inflammatory phenotype in these cells were examined. As shown in Fig. 4D, *CD206, Arginase-1* and *IL-10* were all upregulated by treatment with ox-LDL. However, with exosome administration, these anti-inflammatory markers were significantly upregulated in these macrophages, even compared to the other ox-LDL-treated groups. These results indicated that UC-MSC exosomes may improve the progression of NASH partly by inhibiting ox-LDL-induced macrophage inflammatory phenotypes.

### UC-MSC exosomes reversed PPARα activity in NASH mouse livers and ox-LDL-treated hepatocytes

**Fig. 5:**
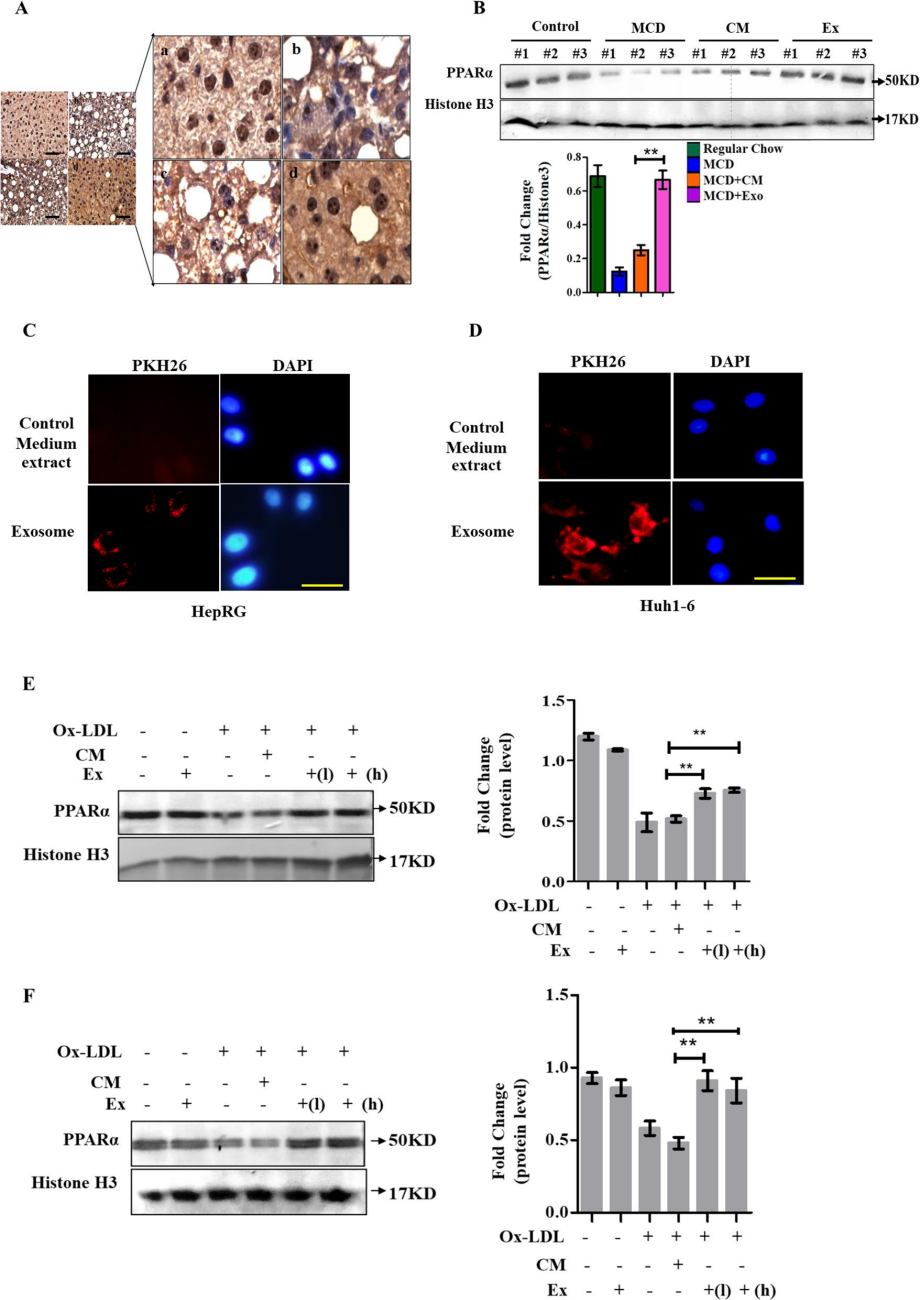
**A**. Immunohistochemical assays were used to detect distribution and location of PPARα in groups of mouse livers. (a) Regular chow; (b) MCD group; (c) MCD with Control Medium (CM) extract intervention group; (d) MCD with hUC-MSC exosomes(Ex) group. The random view to zoom in on the panel shown is on the right. Scale bar = 20 μm. **B**. Western blotting analysis of PPARα in liver tissues in groups of mice. Three samples were selected in each group (upper panel). Protein levels were quantified using ImageJ software and are represented by a histogram (fold change of PPARα/GAPDH, panel below). Full-length blots are presented in Additional file 1: Fig. S3. **C**. PKH-26 labeling of MSC-exo taken up by HepRG cells. Scale bar = 20 μm. **D**. PKH-26 labeling of MSC-exo taken up by Huh1-6 cells. Scale bar = 20 μm. **E**. Western blotting analysis of PPARα in the human liver cell line HepRG under the indicated treatment. Protein levels were quantified using ImageJ software and represented by a histogram (fold change of PPARα/Histone H3). **p < 0.01. Full-length blots are presented in Additional file 1: Fig. S4. **F**. Western blotting analysis of PPARα in the mouse hepatoma carcinoma cell line Huh1–6 under the indicated treatment (fold change of PPARα/Histone H3). Protein levels were quantified using ImageJ software and represented by a histogram. **p < 0.01. Full-length blots are presented in Additional file 1: Fig. S5

Peroxisomal proliferator-activated receptor (PPAR) α, a member of the nuclear receptor family, is considered to be a major operator of lipid metabolic homeostasis in the liver, and inhibition of its expression is directly related to the pathogenesis of NAFLD and NASH. In this study, we explored whether UC-MSC exosomes could affect the expression of PPARα. As shown in Fig. 5A and B, expression of PPARα in the liver tissues was detected. PPARα displayed positive staining in the nuclei of hepatocytes in mice fed a normal diet. However, the expression of PPARα was significantly downregulated in the hepatocytes of MCD diet-fed mice, which could be reversed by hUC-MSC exosomes. Similar results were also obtained by western blot detection in liver tissue homogenate (Fig. 5B). Then, the phagocytosis of liver parenchymal cells exposed to exosomes was detected. The ability of both human and mouse liver parenchymal cell lines to take up exosomes was tested. As shown in Fig. 5C and D, exosomes could be taken up by both the human liver cell line HepRG and the mouse liver cell line Huh1-6 in one hour. Then, PPARα in these two types of cell lines was detected by western blotting. As shown in Fig. 5E and F, PPARα was downregulated in both cell lines, which could be reversed by UC-MSC exosome treatment (*p* < 0.01). Human liver cells were more sensitive to exosomes, since low-dose (50 ng) intervention led to a reversal of PPARα expression (Fig. 5E). For Huh1-6 mouse liver cells, exosomes increased PPARα at a relatively high dose (200 ng), as shown in Fig. 5F. These results indicated that exosomes may improve the progression of NASH not only by inducing macrophage anti-inflammatory functions but also by affecting PPARα expression.

## Discussion

Nonalcoholic steatohepatitis (NASH) is an advanced form of nonalcoholic fatty liver disease (NAFLD). Currently, no pharmacological therapies have been approved for NASH, and most patients improve NASH progression by lifestyle modifications to reduce weight and enhance lipid metabolism. However, according to statistics, NAFLD is projected to affect 33.5% of the adult population by 2030, of which 27% will suffer from NASH [26, 27]. Therefore, efforts are still needed not only to understand the mechanisms of disease progression but also to establish a probable therapy. Over the past decade, attempts have been made to use mesenchymal stem cells (MSCs) to treat nonalcoholic steatohepatitis. Intravenous administration of bone marrow mesenchymal stem cells (BMSCs) and pluripotent MSCs have been proven to prevent metabolic syndrome in nonalcoholic steatohepatitis mice, including HFD-induced steatosis, decreases in plasma liver enzymes, inflammatory cell infiltration and hepatic triglyceride accumulation [28–30]. In addition, human adipose-derived MSCs have been used to treat high-fat diet-induced obese mice, which yielded a reversal of the pathogenic changes in obesity-related syndromes [31]. For umbilical cord mesenchymal stem cells (UC-MSCs), the possible therapeutic effect has been examined on the NAFLD of type 2 diabetic db/db mice. UC-MSC administration alleviated hepatic functional injury and attenuated hepatic steatosis. Additionally, hepatic lipid accumulation was reduced [19]. Previous studies have demonstrated the regulatory function of MSCs in metabolic syndrome and liver protection, indicating that MSCs are potential candidates for the treatment of nonalcoholic steatohepatitis.

However, studies have shown that MSCs achieve a therapeutic effect in vivo via paracrine action. Recently, studies have indicated that extracellular vesicles (EVs) secreted by hUC-MSCs, which are known as exosomes, contribute to the therapeutic potency of MSCs by transporting paracrine factors during tissue damage repair and immune regulation [32–34]. In this study, our data confirmed that exosomes derived from human umbilical cord MSCs could improve NASH progression in an MCD-induced mouse model. Moreover, our results demonstrated that MSC exosomes could significantly reduce serum alanine transaminase (ALT) and aspartate aminotransferase (AST) activity and the liver histology NAS score, with lower inflammation factor levels than other MCD mice. In a previous study, MSC-derived exosome intervention yielded beneficial effects in various animal models of liver disease, including acute liver injury, liver fibrosis and hepatocellular carcinoma (HCC) [19, 35]. Exosomes have characteristic advantages over hUC-MSCs: the diameter is smaller, the content is less complex, and the exosomes are easier to produce and store. Finally, exosomes could potentially avoid some of the regulatory issues. Therefore, hUC-MSC-derived exosomes may represent a new ideal therapeutic tool for NASH.

To date, the pathogenesis of nonalcoholic steatohepatitis is still not clear, but macrophages are known to be involved in this process. It has been suggested that in NAFLD, macrophages with a proinflammatory phenotype tend to contribute to disease severity. Macrophages are components of the immune system. Once the hepatocytes were damaged, the proinflammatory factors IL-1β and IL-18 were released, which induced a macrophage inflammatory phenotype. These macrophages sequentially produce IL-6, TNF-α and a large amount of IL-1β, promoting the inflammatory response of the liver. In MCD mouse livers, M1-polarized macrophages exhibit an increased tendency toward steatosis and hepatic inflammation [36, 37]. Consistent with our findings, mice that were fed an MCD diet showed upregulated *TNF-α, IL-6* and *IL-1β* in plasma compared with those fed a normal control diet, while exosome administration decreased these cytokines in the plasma. UC-MSC-derived exosomes also upregulated *CD206, IL-10* and *Arginase-1*, indicating that macrophages were potentially induced to M2 polarization. In vitro experiments showed that exosomes also downregulated ox-LDL-induced inflammatory factor production in a mouse macrophage cell line, which indicated that a potentially important target for umbilical cord mesenchymal cell-derived exosomes to ameliorate nonalcoholic steatohepatitis is macrophages. We suspected that exosomes of xenogenic origin could improve disease progression in mice with NASH. Exosomal endocytosis experiments showed that even human-derived exosomes could be efficiently taken up within 1 h by both mouse macrophages and liver parenchymal cells. Therefore, the mouse phenotype could be affected by nucleic acids and proteins in exosomes, even in different species.

Furthermore, NASH is considered to be a continuum of metabolic pathogenesis, which is now widely accepted to result from liver lipotoxicity. In the development of NASH, liver cells are killed by lipotoxicity, leading to inflammatory death [23]. PPARα was the first discovered isotype of the peroxisome proliferator-activated receptors (PPARs) and was named after its ability to induce peroxisome proliferation in rodents. PPARs modulate gene transcription by binding to inflammatory transcription factors, such as NF-κB, AP-1, and STATs, to prevent protein binding with response elements. PPARα agonists were also reported to induce an inhibitor of NF-κB in hepatocytes and prevent its translocation from the cytoplasm to the nucleus. A strong correlation between metabolic syndrome and dysregulation of hepatic PPARs has been proven in patients suffering from NASH, so PPARs are considered an essential target for drug intervention, and their agonists are potential therapeutic agents for NASH [38–41]. In this study, exosomes derived from umbilical cord mesenchymal stem cells were taken up by human and murine liver cells in vitro, leading to the reversal of PPARα transcription, which was downregulated under oxidative low-density lipoprotein cholesterol treatment. This new observation also partially reveals the potential mechanism of exosomes in protecting against NASH development. Moreover, previous evidence suggested that PPARα inhibited the proinflammatory phenotype of macrophages by working with STAT6 at the transcriptional level. Our research showed that UC-MSC exosomes could enhance PPARα levels, which may partly explain the decreases in inflammatory factors in MCD-induced NASH mouse liver tissue and the macrophage anti-inflammatory phenotype in vitro [42]. However, due to the complexity of exosome components and individual differences in the hUC-MSC origin, how PPARα gene transcription is regulated by hUC-MSC exosomes in hepatocytes in vitro has not been defined.

## Conclusion

In summary, hUC-MSC exosomes alleviate MCD-induced NASH in mice by regulating the anti-inflammatory phenotype of macrophages and by reversing PPARα protein expression in liver cells. These results reveal that hUC-MSC-derived exosomes hold great potential in hMSC-related NASH therapy, and they are safer and easier to manipulate to control cell-free treatments. Further studies need to focus on understanding the complete mechanism of this complex paracrine communication system to optimize this anti-inflammatory cell-free therapy method for protecting against nonalcoholic steatohepatitis-related end-stage liver disease.

## Supplementary Information

**Additional file 1**: Corresponding uncropped full-length gels and blot in the supplementary file.

## Data Availability

None.

## Abbreviations

hUC-MSCs: Human umbilical cord mesenchymal stem cells
NASH: Nonalcoholic steatohepatitis
PPARα: Peroxisome proliferator-activated receptor α
Ox-LDL: Oxidized low-density lipoprotein
ALT: Alanine transaminase
NAFLD: Nonalcoholic fatty liver disease
MCD: Methionine–choline-deficient
FBS: Fetal bovine serum
TEM: Transmission electron microscopy
H&E: Hematoxylin and eosin
ELISA: Enzyme-linked immunosorbent assay
CM: Control medium
EV: Extracellular vesicle

## References

[1] Samira R, Bahareh A, Seyed HM (2020). Umbilical cord-derived mesenchymal stem cells in neurodegenerative disorders: from literature to clinical practice. Regen Med.

[2] Xu W-X, He H-L, Pan S-W, Chen Y-L, Zhang M-L, Zhu S (2019). Combination treatments of plasma exchange and umbilical cord-derived mesenchymal stem cell transplantation for patients with hepatitis b virus-related acute-on-chronic liver failure: a clinical trial in China. Stem Cells Int..

[3] Gomes A, Coelho P, Soares R, Costa R (2021). Human umbilical cord mesenchymal stem cells in type 2 diabetes mellitus: the emerging therapeutic approach. Cell Tissue Res..

[4] Mebarki M, Abadie C, Larghero J, Cras A (2021). Human umbilical cord-derived mesenchymal stem/stromal cells: a promising candidate for the development of advanced therapy medicinal products. Stem Cell Res Ther.

[5] Pajarinen J, Lin T, Gibon E, Kohno Y, Maruyama M, Nathan K (2019). Mesenchymal stem cell-macrophage crosstalk and bone healing. Biomaterials.

[6] Lopatina T, Gai C, Deregibus MC, Kholia S, Camussi G (2016). Cross talk between cancer and mesenchymal stem cells through extracellular vesicles carrying nucleic acids. Front Oncol.

[7] Abbaszadeh H, Ghorbani F, Derakhshani M, Movassaghpour A, Yousefi M (2020). Human umbilical cord mesenchymal stem cell-derived extracellular vesicles: a novel therapeutic paradigm. J Cell Physiol.

[8] Keshtkar S, Azarpira N, Ghahremani MH (2018). Mesenchymal stem cell-derived extracellular vesicles: novel frontiers in regenerative medicine. Stem Cell Res Ther.

[9] Ha DH, Kim H-K, Lee J, Kwon HH, Park G-H, Yang SH (2020). Mesenchymal stem/stromal cell-derived exosomes for immunomodulatory therapeutics and skin regeneration. Cells.

[10] Elahi FM, Farwell DG, Nolta JA, Anderson JD (2020). Preclinical translation of exosomes derived from mesenchymal stem/stromal cells. Stem Cells.

[11] Reza-Zaldivar EE, Hernández-Sapiéns MA, Minjarez B, Gutiérrez-Mercado YK, Márquez-Aguirre AL, Canales-Aguirre AA (2018). Potential effects of MSC-derived exosomes in neuroplasticity in alzheimer's disease. Front Cell Neurosci.

[12] Harrell CR, Markovic BS, Fellabaum C, Arsenijevic A, Djonov V, Arsenijevic N (2018). Therapeutic potential of mesenchymal stem cell-derived exosomes in the treatment of eye diseases. Adv Exp Med Biol.

[13] Younossi ZM (2019). Non-alcoholic fatty liver disease—a global public health perspective. J Hepatol.

[14] Bedossa P (2017). Pathology of non-alcoholic fatty liver disease. Liver Int.

[15] Farzanegi P, Dana A, Ebrahimpoor Z, Asadi M, Azarbayjani MA (2019). Mechanisms of beneficial effects of exercise training on non-alcoholic fatty liver disease (NAFLD): Roles of oxidative stress and inflammation. Eur J Sport Sci.

[16] Cobbina E, Akhlaghi F (2017). Non-alcoholic fatty liver disease (NAFLD) - pathogenesis, classification, and effect on drug metabolizing enzymes and transporters. Drug Metab Rev.

[17] Cole BK, Feaver RE, Wamhoff BR, Dash A (2018). Non-alcoholic fatty liver disease (NAFLD) models in drug discovery. Expert Opin Drug Discov.

[18] Sumida Y, Yoneda M (2018). Current and future pharmacological therapies for NAFLD/NASH. J Gastroenterol.

[19] Chenxia Hu, Zhongwen Wu, Li L (2020). Mesenchymal stromal cells promote liver regeneration through regulation of immune cells. Int J Biol Sci.

[20] Bing Li Yu, Cheng SY, Zang Li, Yin Y, Liu J (2019). Human umbilical cord-derived mesenchymal stem cell therapy ameliorates nonalcoholic fatty liver disease in obese type 2 diabetic mice. Stem Cells Int.

[21] Phinney DG, Prockop DJ (2007). Concise review: mesenchymal stem/multipotent stromal cells: the state of transdifferentiation and modes of tissue repair—current views. Stem Cells.

[22] Furlani D, Ugurlucan M, Ong L, Bieback K, Pittermann E, Westien I (2009). Is the intravascular administration of mesenchymal stem cells safe? Mesenchymal stem cells and intravital microscopy. Microvasc Res.

[23] Watanabe T, Tsuchiya A, Takeuchi S (2020). Development of a non-alcoholic steatohepatitis model with rapid accumulation of fibrosis, and its treatment using mesenchymal stem cells and their small extracellular vesicles. Regen Ther.

[24] Che Y, Shi X, Zhong X, Zhang Y, Si R, Li Y, Shi Y (2020). Resveratrol prevents liver damage in MCD-induced steatohepatitis mice by promoting SIGIRR gene transcription. J Nutr Biochem.

[25] Kleiner DE, Brunt EM, Van Natta M (2005). Design and validation of a histological scoring system for nonalcoholic fatty liver disease. Hepatology.

[26] Younossi ZM, Blissett D, Blissett R, Henry L, Stepanova M, Younossi Y (2016). The economic and clinical burden of nonalcoholic fatty liver disease in the United States and Europe. Hepatology.

[27] Younossi ZM, Golabi P, De Avila L, Paik JM, Srishord M, Fukui N (2019). The global epidemiology of NAFLD and NASH in patients with type 2 diabetes: a systematic review and meta-analysis. J Hepatol.

[28] Ezquer M, Ezquer F, Ricca M (2011). Intravenous administration of multipotent stromal cells prevents the onset of non-alcoholic steatohepatitis in obese mice with metabolic syndrome. J Hepatol.

[29] Bi Y, Guo X, Zhang M, Zhu K, Shi C, Fan B (2021). Bone marrow derived-mesenchymal stem cell improves diabetes-associated fatty liver via mitochondria transformation in mice. Stem Cell Res Ther.

[30] Ezquer M, Ezquer F, Ricca M, Allers C, Conget P (2011). Intravenous administration of multipotent stromal cells prevents the onset of non-alcoholic steatohepatitis in obese mice with metabolic syndrome. J Hepatol.

[31] Seki A, Sakai Y, Komura T, Nasti A, Yoshida K, Higashimoto M (2013). Adipose tissue-derived stem cells as a regenerative therapy for a mouse steatohepatitis-induced cirrhosis model. Hepatology.

[32] Shi Y, Wang Y, Li Q, Liu K, Hou J, Shao C (2018). Immunoregulatory mechanisms of mesenchymal stem and stromal cells in inflammatory diseases. Nat Rev Nephrol.

[33] Qi K, Li Na, Zhang Z, Melino G (2018). Tissue regeneration: the crosstalk between mesenchymal stem cells and immune response. Cell Immunol.

[34] Byrnes D, Masterson CH, Artigas A, Laffey JG (2021). Mesenchymal stem/stromal cells therapy for sepsis and acute respiratory distress syndrome. Semin Respir Crit Care Med.

[35] Lou G, Chen Z, Zheng M, Liu Y (2017). Mesenchymal stem cell-derived exosomes as a new therapeutic strategy for liver diseases. Exp Mol Med.

[36] Schuppan D, Surabattula R, Wang XY (2018). Determinants of fibrosis progression and regression in NASH. J Hepatol.

[37] Lefere S, Tacke F (2019). Macrophages in obesity and non-alcoholic fatty liver disease: crosstalk with metabolism. JHEP Rep.

[38] Boeckmans J, Natale A, Rombaut M, Buyl K, Rogiers V, De Kock J (2019). Anti-NASH drug development hitches a lift on PPAR agonism. Cells.

[39] Westerouen Van Meeteren MJ, Drenth JPH, Tjwa ETTL (2020). Elafibranor: a potential drug for the treatment of nonalcoholic steatohepatitis (NASH). Expert Opin Investig Drugs.

[40] Jain MR, Giri SR, Bhoi B, Trivedi C, Rath A, Rathod R (2018). Dual PPARα/γ agonist saroglitazar improves liver histopathology and biochemistry in experimental NASH models. Liver Int.

[41] Odegaard JI, Ricardo-Gonzalez RR, Eagle AR, Vats D, Morel CR, Goforth MH (2008). Alternative M2 activation of Kupffer cells by PPAR delta ameliorates obesity-induced insulin resistance. Cell Metab.

[42] Luke A, O’Neill J, Kishton RJ, Rathmell J (2016). A guide to immunometabolism for immunologists. Nat Rev Immunol.

