# Supplementary figures and images for: Human umbilical cord mesenchymal stromal cell-derived exosomes protect against MCD-induced NASH in a mouse model
Source: Stem Cell Res Ther. 2022 Nov 12;13:517. doi: 10.1186/s13287-022-03201-7 (PMC9652856; doi:10.1186/s13287-022-03201-7)

**Supplementary Figures**

**Figure1**


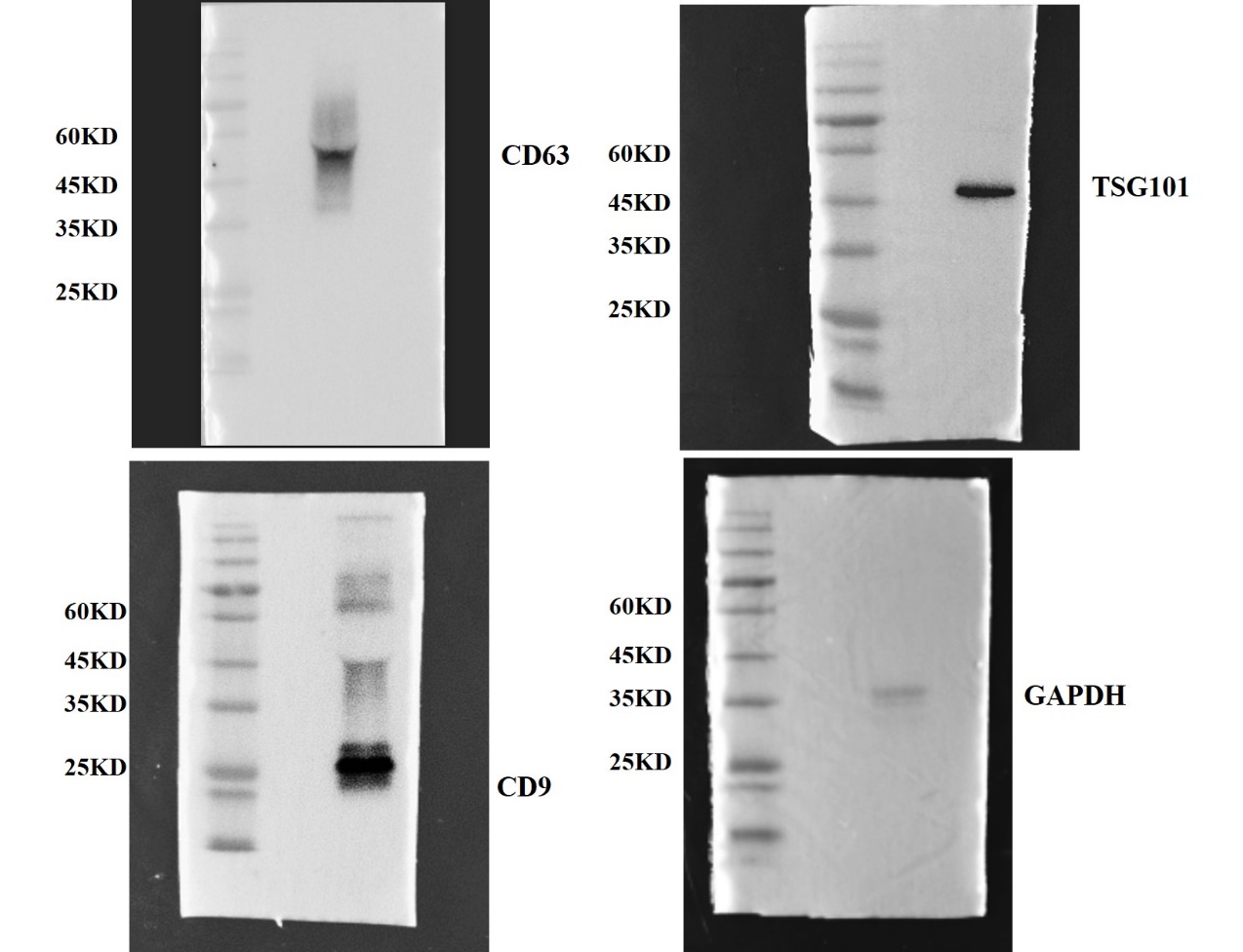


**Figure2**

**
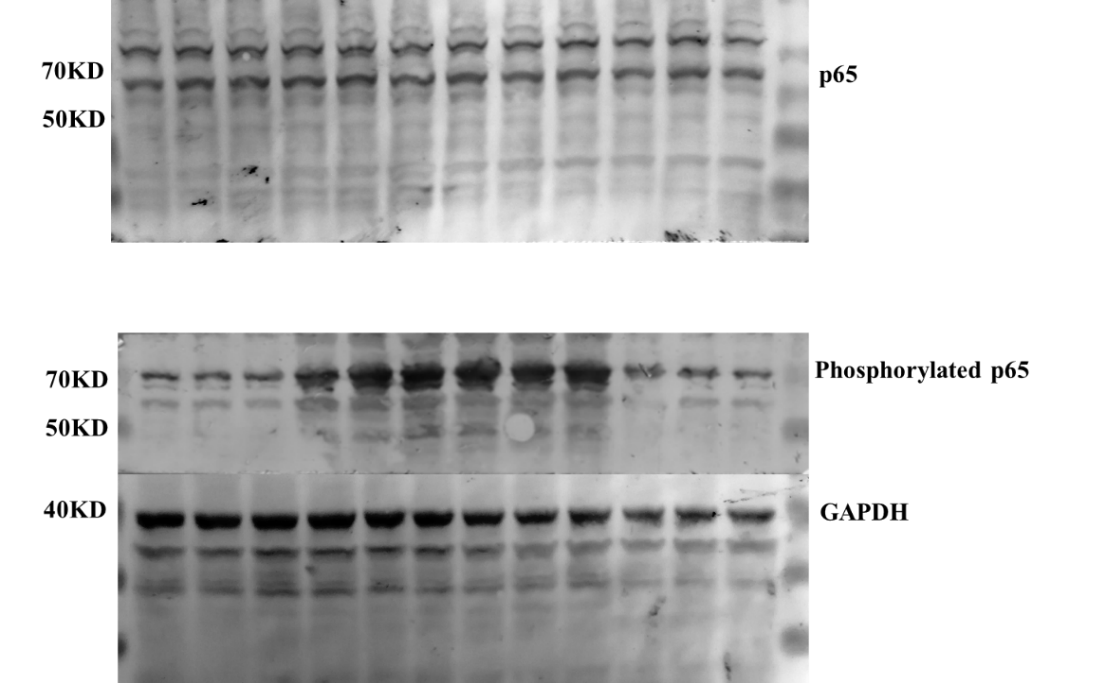
**

**Figure3**

**
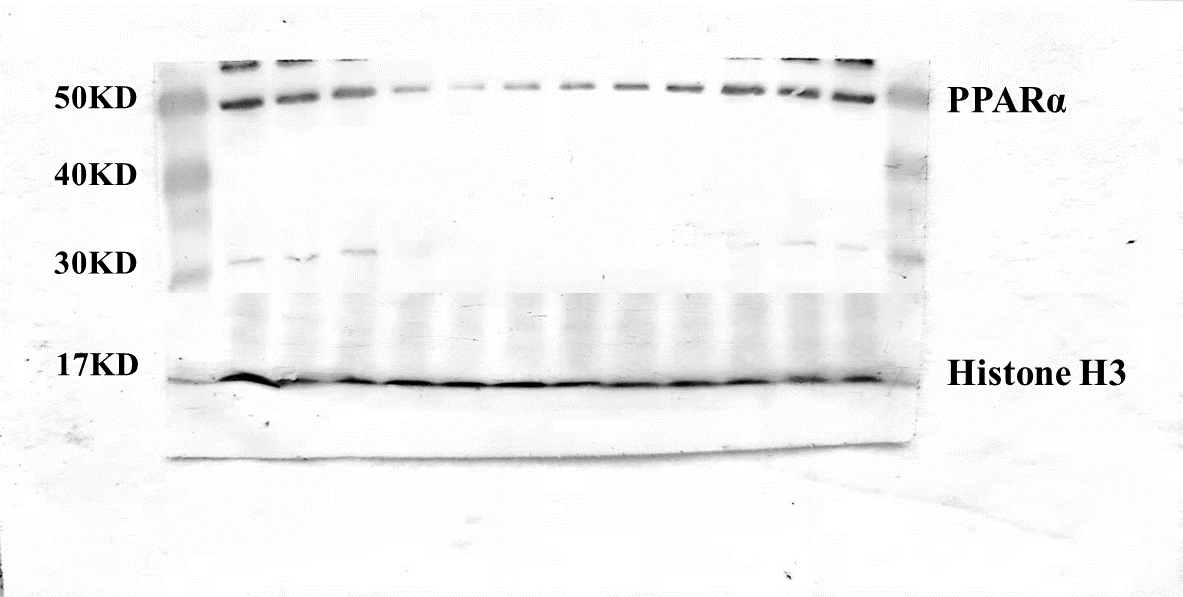
**

**Figure4**

**
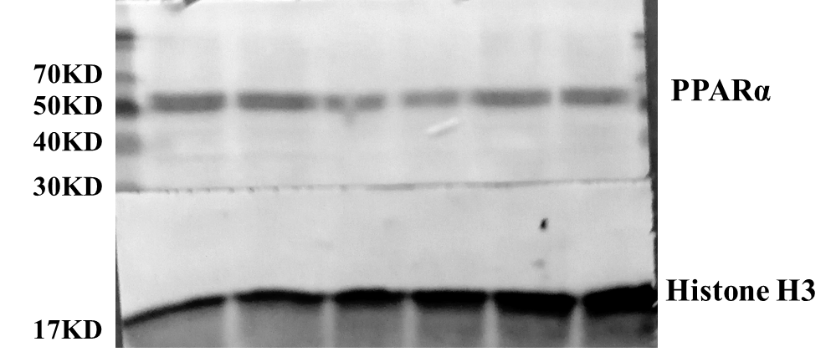
**

**Figure5**

**
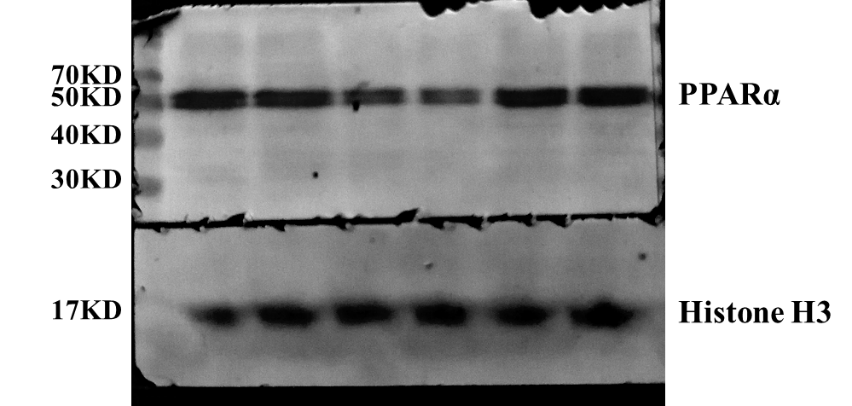
**

Supplement: Supplementary file 1 — Additional file 1: Corresponding uncropped full-length gels and blot in the supplementary file. [file 13287_2022_3201_MOESM1_ESM.docx]
